# Human Disturbances, Habitat Characteristics and Social Environment Generate Sex-Specific Responses in Vigilance of Mediterranean Mouflon

**DOI:** 10.1371/journal.pone.0082960

**Published:** 2013-12-30

**Authors:** Stéphanie Benoist, Mathieu Garel, Jean-Marc Cugnasse, Pierrick Blanchard

**Affiliations:** 1 Office National de la Chasse et de la Faune Sauvage, Centre National d'Etudes et de Recherche Appliquée sur la Faune de Montagne, Gières, France; 2 Office National de la Chasse et de la Faune Sauvage, Direction des Etudes et de la Recherche, Toulouse, France; 3 Université de Toulouse, CNRS, ENFA; UMR 5174, Laboratoire Evolution et Diversité Biologique, Toulouse, France; Institut Pluridisciplinaire Hubert Curien, France

## Abstract

In prey species, vigilance is an important part of the decision making process related to predation risk effects. Therefore, understanding the mechanisms shaping vigilance behavior provides relevant insights on factors influencing individual fitness. We investigated the role of extrinsic and intrinsic factors on vigilance behavior in Mediterranean mouflon (*Ovis gmelini musimon*×*Ovis* sp.) in a study site spatially and temporally contrasted in human pressures. Both sexes were less vigilant in the wildlife reserve compared to surrounding unprotected areas, except for males during the hunting period. During this period, males tended to be less strictly restricted to the reserve than females what might lead to a pervasive effect of hunting within the protected area, resulting in an increase in male vigilance. It might also be a rutting effect that did not occur in unprotected areas because males vigilance was already maximal in response to human disturbances. In both sexes, yearlings were less vigilant than adults, probably because they traded off vigilance for learning and energy acquisition and/or because they relied on adult experience present in the group. Similarly, non-reproductive females benefited of the vigilance effort provided by reproductive females when belonging to the same group. However, in the absence of reproductive females, non-reproductive females were as vigilant as reproductive females. Increasing group size was only found to reduce vigilance in females (up to 17.5%), not in males. We also showed sex-specific responses to habitat characteristics. Females increased their vigilance when habitat visibility decreased (up to 13.8%) whereas males increased their vigilance when feeding on low quality sites, i.e., when concomitant increase in chewing time can be devoted to vigilance with limited costs. Our global approach was able to disentangle the sex-specific sources of variation in mouflon vigilance and stressed the importance of reserves in managing and conserving wild sheep populations.

## Introduction

Predators may impact demography of their preys both through lethal direct effects, and lethal and non-lethal indirect effects [1]–[4]. Indirect effects depend on how predators cause adaptive shifts in prey behavior or life history allocation [5], [6]. The costs arising from behavioral changes shaped by predation pressureare known as risk effects [5]–[7]. Previous studies have shown that risk effects can impact prey dynamics even more than the direct mortality due to killing by predators [6], [8]. For instance, it has been shown in grey partridges (*Perdix perdix*) that the scanning of the surroundings in order to detect predators arose at the expense of feeding under certain circumstances and, accordingly, may impact fitness over the long term [9].

Many behavioural decisions are affected by the risk of predation. Among them, vigilance is probably the most widely and successfully studied due to its central role in the detection and avoidance of predators [10]. As such, the time an animal spends scanning the surroundings has been used as a proxy of its perception of risk. A better understanding of the factors shaping vigilance behavior should therefore help to provide insight into spatial, temporal and inter-individual variation in risk effects, and thus in mechanisms causing fitness variation among individuals [11]. Whereas our knowledge has considerably improved over the last decade on independent factors influencing vigilance behavior, global approaches investigating simultaneously (e.g., [12]) and interactively (e.g., [13]) all important components expected to influence vigilance behavior (spatial, temporal and individual variation) have focused less attention.

Many factors are known to influence risk perception and thus, vigilance behavior. Among them, human disturbances [14], [15], characteristics of the environment, such as habitat visibility [9], [16]–[18] and group size, through dilution [19]–[21] and many-eyes effects [22], [23], have been repeatedly reported. Intrinsic characteristics (sex, age and reproductive status) of the focal individual have also been proved to shape vigilance patterns. For instance, females with young are known to be highly vigilant in order to maximise offspring survival [24]–[28], especially during the birth period because of the vulnerability of their young [27]. Older individuals can also be expected to be more vigilant than younger conspecifics [29] that faced high energy requirement during their first years of life (e.g., [30] in chamois *Rupicapra rupicapra*) and should in turn trade vigilance for energy acquisition [31]. Intrinsic characteristics of conspecifics in the group, possibly in interaction with group size, are also expected to affect vigilance patterns [32], [33]. For instance, the lower vigilance of young individuals could be balanced by relying on adult experience present in the group which have acquired skills in allocating their time efficiently between potentially conflicting activities such as foraging, avoiding predators and interacting with conspecifics (reviewed in [34]). Similarly, non-reproductive females and males probably benefit from the higher investment in vigilance by reproducing females to reduce their own vigilance when foraging in the same group [32].

Here, we aimed at using a global approach to investigate simultaneously and interactively the role of important components expected to influence vigilance behavior in male and female Mediterranean mouflon (*Ovis gmelini musimon*×*Ovis* sp.). Study site spatially contrasted a central Wildlife Reserve (WR), without hunting and with restricted recreational activities, surrounded by unprotected areas (hereafter called UA for unprotected areas) with marked spatio-temporal variation in recreational activities and high hunting pressure occurring from 1 September to the end of February [35], [36]. How protected areas influence the behavior of the targeted species still remains a challenging question for population biologists (e.g., [37], [38]). We took advantage of our spatial (WR vs. UA) and temporal (hunting vs. non-hunting period) variation in term of human pressure to address this question and assessed simultaneously the relative roles of extrinsic (habitat visibility, group size and composition) and intrinsic (age, reproductive status) factors known to shape vigilance patterns (see Table 1 for studied hypotheses). Among specific hypotheses, we expected a lower level of vigilance of mouflon located in the WR compared to animals located in the UA, and that the magnitude of such an effect increases during the hunting period [15], [37]. Because some females in our population are horned and may benefit of better defence capabilities than hornless females [39], we included the horn phenotype of females in our analysis expecting hornless females to be more vigilant than horned females. Beyond factors known to influence risk perception, we also expected males to increase their level of vigilance during the rut in a context of social dominance [40]. Lastly, because food characteristics may also impact vigilance patterns [41]–[43], we included patch quality in our analysis and predicted that the expected increase in the requested chewing time in poor feeding habitats should lead to an increase in the amount of time available for vigilance.

**Table 1:** Hypotheses tested and related variables.

Sources of variation in vigilance behavior	Expected effect	Associated variables	Descriptions
Human disturbances			
Hunting period	Vigilance is higher during the hunting period compared to the non hunting period	Hunting	2 levels (March–August without hunting and September–November with hunting)
Area characteristics	Vigilance is higher in UA (recreational activity unrestricted and hunting during part of the year) compared to the WR (restricted recreational activity and no hunting)	Area	2 levels (UA and WR)
Environmental characteristics			
Habitat visibility	Vigilance increases with a decreasing number of visible pixels surrounding animals, i.e., with a decrease in the probability to detect predators (see Methods for more details on computation)	Visibility	Continuous variable ranging from 18.1 pixels to 116.7 pixels
Quality of feeding sites	Vigilance increases when feeding on low quality feeding sites because longer chewing time can be devoted to vigilance without additional cost	Feeding	2 levels (high quality or low quality feeding sites)
Individual and social characteristics			
Presence of horn in females	Horned females are less vigilant than hornless females due to better defence capabilities	Horn	2 levels (with or without horns)
Lambing periods in females	Vigilance is higher during the lambing period (where >80% of females reproduce) than later in the year to maximise offspring survival when lambs are the more vulnerable	Lambing	2 levels (March–June or July–November)
Onset of rut in males	Vigilance is higher for males during rut than in non reproductive period as a behavioural response to social dominance	Onset of rut	2 levels (March–September or October–November)
Reproductive status	Females with lamb are more vigilant than non reproductive females to maximise offspring survival	Repro	2 levels (female with lamb or female without lamb)
Reproductive composition of the group	Because non reproductive females rely on the higher investment in vigilance provided by reproductive females, they can decrease their vigilance when foraging in mixed reproductive status female groups.	Repro compF (for females)	3 levels (female with lamb or female without lamb in a reproductive group or in a non-reproductive group)
	Males foraging with reproductive females decrease their vigilance as compared to males foraging with barren females because they rely of the extra investment in vigilance provided by reproductive females.	Repro compM (for males)	2 levels (male in a reproductive group or not)
Age	Yearlings are less vigilant than adults because of the need to ensure high food intake in the first years of life at the expense of other behavioral activities	Age	2 levels (yearlings or adults)
Age composition of the group	When belonging to mixed groups (adults+yearlings), yearlings benefit of higher vigilance of adults to be less vigilant than when belonging to juvenile groups (only yearlings)	Age comp	3 levels (yearlings only or yearlings with adults or adults alone)
Group size	Individual vigilance decreases with an increasing group size either as the result of dilution or many-eyes effects	Group size	Continuous variable ranging from 1 to 50 mouflon

## Materials and Methods

### Ethics Statement

Our research protocols have been approved by our National Wildlife Management Agency (French Ministry of Environment). The study was observational (without human disturbance), and involved no invasive method and cruelty to animals. Thus no review from the ethic committee was required in France. Our field study did not involve endangered or protected species. Monitoring was performed within a French National Wildlife Reserve (Office National des Forts and Office National de la Chasse et de la Faune Sauvage) and on lands that are of public access (a very important regional tourist site with more than 300 000 tourists/year in the south part). The project was validated and supported respectively by the Director of the Wildlife Reserve and the elected representatives of the local landowners (SIVOM of Caroux-Espinouse).

### Study area and population

**Figure 1:**
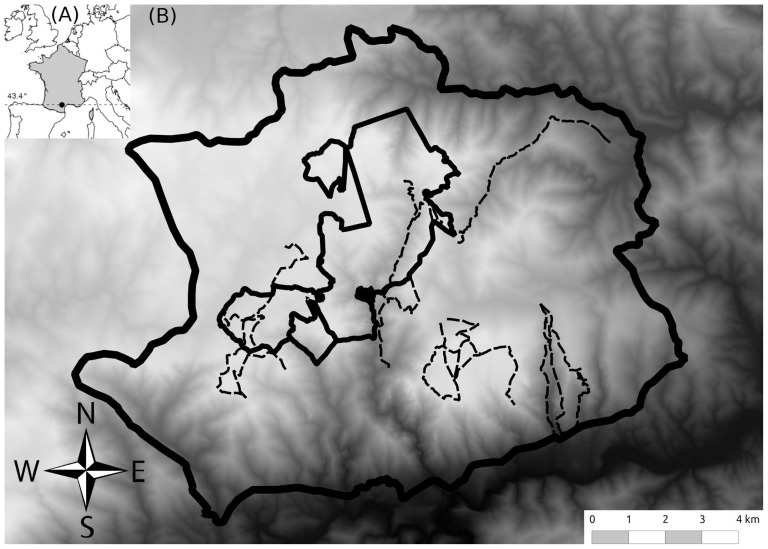
(A) Location of the Caroux-Espinouse massif in France. (B) Digital elevation model (150–1124 m a.s.l.) of the range of the mouflon population in 1998 (thick plain line). Wildlife Reserve (thin plain lines, 1704 ha) and the 5 transects sampled (dotted lines) are also reported.

The population of mouflon inhabits the Caroux€Espinouse massif (43°38′N, 2°58′E; elevation 150–1124 m a.s.l.) in the Southern border of the Massif Central, in Southern France (Fig. 1A). The massif consists of high plateaus alternating with deep valleys (Fig. 1B). Vegetation is an irregular mosaic of beech, chestnut, coniferous, ever-green oak with open areas dominated by moorlands of heather and broom. Except in the WR (1704 ha, created in 1956; Fig. 1B), hunting occurred from 1 September to the end of February for mouflon, wild boar (*Sus scrofa*), and roe deer (*Capreolus capreolus*) and has been shown to influence population dynamics of the target species [44]. Hunters harvested the same number of female and male mouflon. Stalking (usually for trophy hunting of the largest horned males; [36]) and driven hunting were the two most common hunting practices. Stalking involved the presence of a group of 2–4 humans whereas wild boar hunting involved the presence of numerous hunters and domestic dogs [45]. Driven hunting was primarily performed for wild boar hunting during which male and female mouflon were also harvested. Human activities were restricted in WR (no hunting, hiking permitted only on one trail across the WR and on two ones bordering it) limiting human disturbances as compared to most parts of the massif (UA) where hunting [36] and marked spatio-temporal variation in recreational activities occurred [35].

Roe deer and wild boar (at low density comparatively to the mouflon population) were the two other free-ranging ungulates inhabiting this massif. Mouflon had no natural predators in the study area, except for free-roaming dogs, golden eagles (*Aquila chrysaetos*) and red foxes (*Vulpes vulpes*) that may occasionally predate newborns and sick mouflon [46], [47].

Most births occurred in April, but the lambing season ranged from late March to June ([48]; Table 1) during which a high proportion of females reproduce every year (>80%, [49]). The gestation period is of 5 months, implying that ewes are fertilized between late October and early January [49], but mouflon rams roam from one female group to another and exhibit courtship behavior from the beginning of October ([50]; our data). Accordingly, the whole rutting period may be considered to stretch from early October to early January (Table 1).

Mouflon is classified as grazer (*sensu* [51]) including herbaceous species in a large proportion of its diet (40–50% of grasses in our population; [52]; see [53] for a review). Accordingly, mouflon fed primarily in open habitats composed of meadows/pasture (*Brachypodium sylvaticum*, *Festuca rubra*, *F. paniculata*, *F. ovina*, *Carex* sp.) and moorlands of heather (*Erica cinerea*, *Calluna vulgaris* and *Carex pilulifera*) and broom (*Genista pilosa*, *G. anglica*, *Cytisus oromediterraneus*, and *C. scoparius*) that offer the highest abundance of resources [54]. We restricted our analysis to these feeding patches and we investigated the role of habitat quality on vigilance behavior [55]. Specifically, we opposed moorlands (feeding sites of lower quality) and meadows/game cultures (feeding sites of higher quality) because moorlands are dominated by ligneous species of low digestibility for mouflon [52], [54].

### Data collection

Data were simultaneously collected along 5 fixed transects (around 13 km for each; Fig. 1B), walked simultaneously on the entire diurnal period of the day, from 3 March, 1996 to 3 November, 1996 (see [35] for more details). These 5 transects cover 29.5% of the population's range. Data were sampled both during weekends (n = 15) and other weekdays (n = 13) to ensure that all conditions of human disturbances were included. These transects were not selected randomly but chosen to sample the environmental diversity in term of habitat types and human pressures (tourism and hunting) over the study area and to offer the best observational conditions (e.g., offering the largest viewpoints on open areas commonly used by mouflon when feeding; [35]). Spatial coordinates (based on a 125×125 m grid size), group size and group composition in age-sex classes were recorded for each group encountered as well as the habitat used (4 habitats: forest, rock, moorlands and meadow/pasture – our analyses were restricted to the two latter habitats, see “Study area and population”). We chose to distinguish three age categories [56]: lambs, yearlings (1 year old) and adults (>1year old). Because gender was hardly distinguishable in lambs during the first months of life, we restricted the analysis to the two former age classes (Table 1). For each mouflon composing the group, we also recorded individual activity (see below), reproductive status (with or without lamb; lamb being observed suckling and/or following its mother; [57]) and presence of horns for females (25.6% of females observed were horned during the study period).

Seven activity items were identified using scan sampling [58]: feeding, resting, in movement, playing, in rut, in flight and vigilant. The vigilance item corresponds to mouflon with their head raised above shoulder level and scanning their surroundings. It has to be noted that a same animal can be repeatedly seen over several days because mouflon were not individually marked.

### Spatial covariates

We used the spatial coordinates of a group to assign it to the WR or to the UA. We also computed a proxy of habitat visibility experienced by each group of mouflon. We used an elevation raster map (25 m×25 m grid size) and a line-of-sight raster analysis in GRASS [59]. Line-of-sight raster analysis generates a raster output map in which the pixels that are visible from the user-specified observer position are marked with the vertical angle (in degrees) required to see those pixels. Pixels not visible were marked with null value. Elevation layer accounted for the vegetation cover of each pixel (coded open/closed; source: Occupation du sol LR 1999–2006/SIG LR project; www.siglr.org) so that pixels coded as closed habitats were set to null value in the elevation map. Computation was performed using a mouflon height of 1 m above the viewing point's elevation and a maximum distance from the viewing point inside of which the line of sight analysis will be performed of 600 m. Such a map was then used to compute the number of pixels visible from a given pixel. The procedure was repeated for each pixel composing the elevation map to get a layer of visibility. Because mouflon groups were spotted on a 125×125 grid size, we lowered the resolution of the layer of visibility by merging together squares of 5×5 pixels and took the average of the number of visible pixels of the 25 pixels composing the new cells of the grid. We finally computed buffers (i.e., circles centered on group coordinates) to obtain estimates of landscape visibility experienced by each mouflon within its theoretical “home range”. Buffer size was computed as the average home range of 46 mouflon (males = 294 ha, i.e., circles with a radius of 968 m; females = 178 ha, i.e., circles with a radius of 753 m; Marchand et al. unpublished data based on 16 males and 30 females) trapped and fitted with Lotek GPS collars 3300S (revision 2; Lotek Engineering Inc., Carp, Ontario, Canada). Estimates of landscape visibility within males and females buffer corresponded to the average of visible pixels from each pixel included in the buffer.

### Data analysis

We performed sex-specific analysis and tested our hypothesis (see Table 1 for details) using a logistic regression (generalized linear model with binomial distribution and logit link) with vigilance status as the binary dependent variable. We tested for two-way interactions between habitat visibility and group size [13] and between hunting period and protected/unprotected areas. During model selection, we concurrently assessed the effect of variables including characteristics of the animals in the group (“Repro comp. F” and “Age comp.”) against the related variables of the focal animal (“Repro” and “Age”; see Table 1 for details). Because we only included factors for which we had 30 data for each modality, we only used age as a factor with two levels for females (“Age”, see Table 1) and not as a three levels factor (“Age comp.”). Indeed, in females, groups only composed of yearlings occurred very seldom, yearlings commonly belonging to the matrilineal group.

We used backward stepwise selection procedures. We tested successively the main effects of factors and the two-way interactions against the most general model by using likelihood-ratio chi-squares tests [60]. A variable was considered significant when p<0.05. To ensure that we selected the most explanatory variables [61], we also performed the model selection using the Akaike Information Criterion (AIC) with second order adjustment (AICc) to correct for small-sample bias ([62]; see Tables S1, S2). Models with ΔAICc<2 can be considered to be equally supported by the data [62].

When including covariates in a logistic model, the number of observations within each sample unit may become equal to one. This precludes assessment of goodness-of-fit using the standard Pearson chi-squared statistic [63]. We therefore used the Cessie-van Houwelingen goodness of fit test [64] based on smoothing methods to assess the overall fit of the selected models. Similarly, computing coefficient of determination in logistic models is a challenging task which has led to the development of several competing measures [63], [65], [66]. We chose to report the adjusted coefficient of determination (R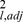) proposed by [66]. For all statistical analyses, we used R version 2.15.3 ([67]; R codes and data available on request from M. Garel).

## Results

A total of 586 groups were observed on feeding zones over 28 days of observations (13 during the week and 15 during the weekend). In total, 539 females (average proportion of animals in vigilance ± SE: 5.75%±1.00%) and 613 males (10.77%±1.25%) were observed.

### Selected models

We identified two different models for males and females. The best model for males (R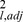 = 0.039, see [68] for a point of comparison of R^2^ values in ecological studies) included the interaction between hunting period and the protection status of the area, and additive effects of age and quality of feeding site (Table 2). As including the age composition of the group instead of the age of the individual only (Table 1) did not provide a better fit (*χ* ^2^ = 1.932, d.f. = 1, p = 0.165), we used the most simple model (“Age” with two modalities). The best model for females (R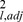 = 0.142) included additive effects of age, habitat visibility, group size, protection status of the area and reproductive composition of the group (Table 3). Although effect of habitat visibility was barely significant (p = 0.059), we kept this effect in the model as it was strongly supported according to AICc (Table S2). The reproductive status of the group explained the probability of being vigilant for females, more than the individual reproductive status of the focal animal (Table 1; 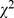 = 3.899, d.f. = 1, p = 0.048). Both for males and females, selected models fitted the data satisfactorily (Cessie-van Houwelingen goodness of fit tests for males: *z* = 0.376, p = 0.707; for females: *z* = 1.470, p = 0.142).

**Table 2:** Generalized linear model (using a logit link) of vigilance probability in mouflon males, Caroux-Espinouse massif, France.

Vigilance Terms	Deviance	DF	p(*χ* ^2^)
Visibility×Group size	0.191	1	0.663
Visibility	0.255	1	0.613
Repro compM	1.564	2	0.458
Group size	0.123	1	0.726
Onset of Rut	1.782	1	0.182
Age comp	6.638	2	0.036
**(Age)**	**4.706**	**1**	**0.030**
**Feeding**	**6.047**	**1**	**0.014**
**Hunting×Area**	**6.406**	**1**	**0.011**

Best model	Coefficient	SE
Intercept	−3.435	1.095
Yearling	−0.833	0.419
Feeding sites of low quality	1.867	1.024
Non-hunting season	−2.324	1.083
UA	−0.266	0.471
Non-hunting season×UA	2.344	1.126

The analysis of deviance table (i.e., difference of deviances between successive nested models) gives the effects of age, quality of feeding sites, mating season, group size, group composition, average visibility in the home range, hunting, area and 2 two-ways interactions on vigilance probability (see Table 1; Full model: Age comp+Repro compM+Onset of Rut+Feeding+Hunting×Area+Visibility×Group size). Variables within brackets were evaluated concurrently to the preceding related variable (see Methods for details). Parameter values with its standard error are given for the best model (significant terms in bold). DF, degrees of freedom and SE, standard error.

**Table 3:** Generalized linear model (using a logit link) of vigilance probability in mouflon females, Caroux-Espinouse massif, France.

Vigilance Terms	Deviance	DF	p(*χ* ^2^)
Horn	0.114	1	0.736
Visibility Group size	0.149	1	0.699
Feeding	1.253	1	0.263
Lambing	2.071	1	0.150
Hunting Area	2.027	1	0.155
Hunting	1.518	1	0.218
**Visibility**	**3.556**	**1**	**0.059**
**Age**	**4.148**	**1**	**0.042**
**Group size**	**4.606**	**1**	**0.032**
**Repro compF**	**7.472**	**2**	**0.024**
(Repro)	3.573	1	0.059
**Area**	**16.428**	**1**	**<0.001**

Best model	Coefficient	SE
Intercept	−3.557	0.873
Yearling	−16.780	1672*
Female without lamb in a reproductive group	−16.830	1750*
Female with lamb	0.482	0.473
UA	2.474	0.773
Visibility	−0.015	0.008
Group size	−0.091	0.054

SEs were not meaningful here because the fitted probabilities were extremely close to zero ([60]; pgs. 197–198).

The analysis of deviance table (i.e., difference of deviances between successive nested models) gives the effects of age, quality of feeding sites, presence of horns, group size, lambing season, group composition, average visibility in the home range, hunting, area and 2 two-ways interactions on vigilance probability (see Table 1; Full model: Age+Repro compF+Lambing+Feeding+Horn+Hunting×Area+Visibility×Group size). Variables within brackets were evaluated concurrently to the preceding related variable (see Methods for details). Parameter values with its standard error are given for the best model (significant terms in bold). DF, degrees of freedom and SE, standard error.

For both sexes, models selected using backward stepwise selection corresponded to (*i*) the best models according to AICc (i.e., lowest AICc), (*ii*) were among the simplest models (lower number of parameters), and (*iii*) included all variables that were the best supported among the best models (ΔAICc<2) (Tables S1, S2). We are therefore confident that our approach should be robust and conservative allowing avoiding the inclusion of spurious effects.

Except if mentioned differently, we reported in the following results the predicted probabilities on the scale of the response variable along with their estimated standard errors for the factor levels “adult” (variable “Age”) and “hunting areas” (variable “Area”) for both males and females. In males only, we also reported predicted effect sizes for the factor level “low quality” (variable “Feeding”) and “non-hunting period” (variable “Hunting”). In females only, we reported predicted effect sizes for the factor level “female with lamb” (reproductive composition of the group), for the average visibility (variable “Visibility”) and the average group size (variable “Group size”).

### Human disturbances

**Figure 2:**
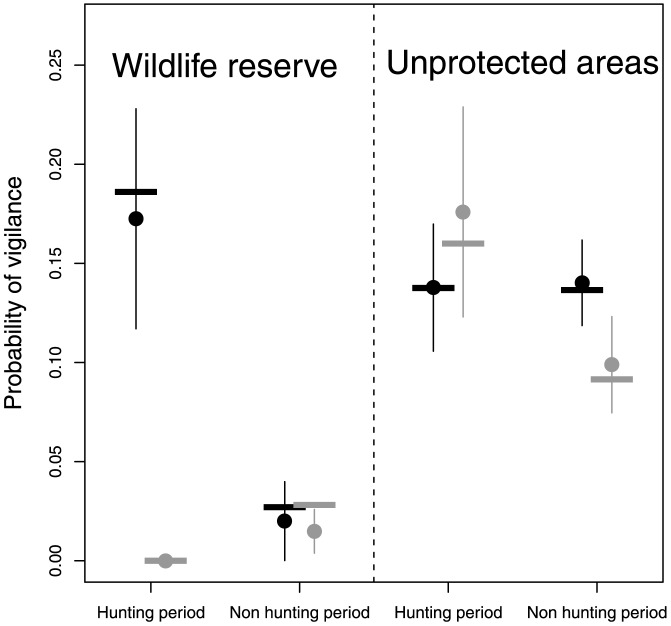
Representation of the best logistic model explaining adult male vigilance according to WR/UA and period of hunting/non hunting (Table 2). Female estimates for a model including the same interaction than males (area status×hunting period) were reported for comparison. Grey circles (± SE) correspond to females and black circles correspond to males. Each circle was drawn along with an horizontal thick line showing the observed proportion of vigilance for the corresponding sex and levels of the factors (observed proportions were computed for the full range of group size and visibility values in females). The predicted probabilities were associated with the level of “low quality feeding sites” for adult males, and with the level of “females with lamb”, the average group size and the average visibility in the home ranges for adult females.

Males and females were much less often vigilant in WR than in UA (adult females: WR: 1.1% 0.8%, n = 97, UA: 11.7% 2.4%, n = 203; Table 2), but only during the non-hunting period for males (Table 3, Fig. 2).

### Environmental characteristics

**Figure 3:**
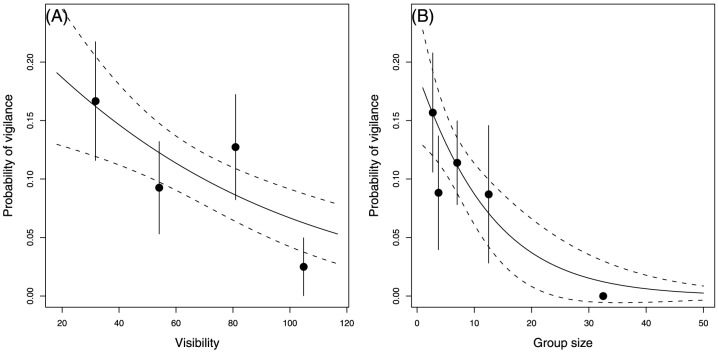
Representation of the covariates ([A]: visibility; [B]: group size) selected in the best logistic model explaining adult female vigilance (Table 3). The fitted logistic models (black lines) as well as their standard errors (dashed lines) were shown. Black circles corresponded to observed proportion (±SE) for a given class of the covariates (sample sizes of classes ranged from 40 to 55 for the covariate “visibility” and from 16 to 79 for the covariate “group size”). The predicted probabilities were associated with the level of “females with lamb” and “no WR”, and for the average group size (A) or the average visibility (B). Observed proportions were computed from a subset of the data including adult females with lamb observed outside the WR, and for the full ranges of group size (A) and habitat visibility values (B).

Females were less vigilant when habitat visibility of their theoretical “home range” (see Methods) increased (slope = −0.015, SE = 0.008; Table 3 and Fig. 3A) whereas no such effect was reported in males (Table 2).

Only males were influenced by the quality of feeding sites (Tables 2 and 3). They were less vigilant when feeding on higher quality pastures (2.4% 2.4%, n = 34) than on lower quality heather and broom moorlands (14.0% 2.2%, n = 203).

### Individual and social characteristics

Group size had a negative effect on female vigilance only (slope = −0.091, SE = 0.054; Table 3 and Fig. 3B). Reproductive periods did not explain vigilance behaviour in neither males nor females (Tables 2 and 3). Age had a significant effect both on the vigilance of males and females, with yearlings (males: 6.6% 2.5%, n = 78; females [“without lamb in a reproductive group”]: 0.0% 0.0%, n = 27) being less vigilant than adults (males: 14.0% 2.2%, n = 249; females [“without lamb in a reproductive group”]: 7.6% 3.1%, n = 78; Tables 2 and 3). Although not providing a better fit than age alone (see “Selected models” section above), age composition of the group suggested that yearling males with adults (4.2% 2.4%, n = 51) had a lower vigilance than in groups of yearlings only (11.6% 5.5%, n = 27) and than males in groups of adults only (14.0% 2.2%, n = 249).

Females without lamb in non-reproductive groups and females with a lamb in reproductive groups had a similar level of vigilance (7.6% 3.1%, n = 78; 11.7% 2.4%, n = 203, respectively), whereas non-reproductive females in a group with reproductive females were never observed vigilant (0.0% 0.0%, n = 38; Table 3). As compared to females, presence of reproductive females in the group did not influence male vigilance (Table 2). Presence of horns did not influence the level of vigilance in females (Table 3).

## Discussion

The increasing focus on how human exploitation affect large herbivores populations [69], [70] strongly supports the need to improve our knowledge on consequences of human activities on animal behaviour. By performing a global approach investigating simultaneously important components expected to influence mouflon vigilance, we were able to disentangle the sex-specific sources of variation in vigilance patterns. We stressed the importance of protected areas acting as a refuge buffering animals against human disturbances. We also emphasized the existence of sex-specific vigilance patterns with males increasing vigilance when feeding on low quality sites and females decreasing vigilance in open areas. Finally, our results suggested that yearling animals traded vigilance for other activities when possible, non-reproductive females benefited from increasing vigilance of reproductive females and group size allow females reducing vigilance costs.

The WR has been created to favour settlement of the mouflon introduced in our study site by protecting them against human activities, with the mid-term perspective of sustainable population harvesting in the surrounding areas. As repeatedly reported across the world, such a measure of protection has been a numerical success [71]. Yet, we still know little about the behavioural mechanisms underlying such successes [72]. Here, we showed that vigilance of mouflon (i.e., a proxy of risk perception and thus of associated risk effects) was low in WR compared to UA. This “reserve effect” suggested that WR buffered females both against recreational and hunting disturbance all year around whereas this effect only held for males outside the hunting period. Absence of a reserve effect for males during the hunting period may be explained by two mutually non-exclusive hypotheses. First, males inhabiting the WR were much less strictly restricted to this area than females, as previously shown using radio tracked animals ([73]–[75]; see also Fig S1, Fig S2). Moreover, most of their movements occurred during the rutting period that is entirely included in the hunting period (Table 1). Among the different forms of philopatry observed in males, part of them move from non-rutting areas located outside the WR to rutting areas located within the WR during the rutting/hunting period [73]. This sex-specific behaviour made males more subject than females during the hunting period to the human pressure of areas surrounding the WR and may explain a pervasive effect of human activities within the WR for males [37]. This result would underline the importance of size of protected areas that should be defined to meet the biological characteristics of the focal species. Second, the rutting period which was thus partly confounded with the hunting period might also explain the pattern reported. During the rut, males typically increase vigilance in order to gather information about mating opportunities and dominance hierarchies [40], leading to a pattern as reported in WR. In UA however, the level of vigilance might have been already too high outside the reproductive period in response to human disturbances to allow males to further increase it in response to reproductive pressures.

Vigilance response of females inhabiting “home range” of lower visibility suggests an increase in risk perception with habitat limiting the probability of detection of an approaching predator (e.g., see [55] for similar results in impala *Aepyceros melampus*). The issue of the effect of reduced visibility is of particular interest for this population that has faced habitat loss over the last past 50 years with open areas decreased by up to 50% [36]. The concurrent decrease in body size (up to 38%) has partly been explained by a shift in diet from herbaceous species to ligneous species [52]. The reduction of high-visibility habitats might also have contributed to affect mouflon growth by constraining animals to spend more time scanning their surroundings when foraging. In this context, management plans for mouflon range improvements, including clear-cutting and range burning, would not be only effective through a positive effect on habitat quality (e.g., [54], [76]) but also on individual behaviour (e.g., [16] in granivorous passerines).

In males, patch quality, more than visibility, influenced vigilance behavior, with an increase in vigilance when foraging on poor quality patches. Because of their larger body mass [36], males are expected to accept lower diet quality than the more selective females [77], spending more time head down looking for more nutritious forages. Moreover, as compared to animals foraging on high-quality plants, animals feeding on poor-quality forages should spend more time chewing so that this time can be devoted to scan their surroundings without any additional cost [41]–[43]. Hence, further studies should include chewing patterns when investigating vigilance behavior and its associated risk effects related to foraging costs [55], [78].

As expected, yearlings were less vigilant than adults. This result may be explained by (*i*) a lack of experience in many aspects of their behavior (foraging, avoiding predators, interaction with conspecifics); (*ii*) their small size, making them harder for predators to detect than adults and thus less constrained to invest in vigilance and/or (*iii*) their greater nutritional needs and, thereby, an increase in foraging in detriment of vigilance [31]. This last hypothesis is the most plausible to explain our results as juveniles, i.e., yearlings in our case, have acquired >1 yr of experience, have relatively large body size compared to adults, but still have high energy requirements. Our results also suggest that this risky behavior would be partly balanced by relying on adults experience, when present in the group. Similarly, we supported the prediction that non-reproductive females benefit of the vigilance effort provided by reproductive females [32], [33], and thus reduced their own investment in vigilance when foraging in the same group. In the absence of reproductive females, barren ewes were as vigilant as reproductive females, thereby mitigating the common assertion that females with young are significantly more vigilant than non-reproductive females (in caribou, *Rangifer tarandus* [24]; in Alpine ibex, *Capra ibex ibex* [79]; in elk, *Cervus elaphus* [27]) and emphasising the importance in such studies to include the reproductive composition of the group instead of relying on the reproductive status of the focal female only.

Although reported as an important factor shaping vigilance patterns in many studies on large herbivores (e.g., [80]), group size appeared only to be a determinant of female vigilance, but not of male vigilance once accounted for other factors (including group size in the best model for males: slope = 0.00002, SE = 0.0256, p = 1). We cannot exclude that this result could arise because of a lack of statistical power to explain residual variation in vigilance behaviour. However, the effect size close to 0 might alternatively support the existence of a real biological mechanism. One explanation could be that the decrease in anti-predatory vigilance when group size (along with the expected detection and dilution effects) increases might have been counter-balanced by a concomitant increase in “social” vigilance [81], supporting the need for further studies investigating the different functions of vigilance.

While mouflon populations on Mediterranean islands undergo strong conservation issues (e.g., [82]), the success of mouflon introduction as a game species all over the world has allowed the development of thriving economical activities based on trophy hunting (e.g., [83]). In this paradoxical context of managing rarity (island populations), quality and abundance (introduced/harvested populations), our work provides managers with valuable information on mouflon behavioral responses to human disturbances, habitat characteristics and the importance of protected areas. Future works should be devoted to quantify the effects on population dynamics (survival, reproduction) of factors shaping vigilance behaviour. This is the essential requirement to ensure the persistence of endangered or economically important populations.

## Supporting Information

Figure S1 **Home ranges (fixed kernel 95% and** ***ad hoc*** **method for smoothing parameter) of 18 females fitted with GPS collars (pink = during hunting period; red = during non-hunting period).** Plain lines correspond to the Wildlife Reserve (WR). As in the WR, hunting was prohibited within the area delimited by a dashed line. However, this area was not considered in the analysis as a protected one because all other recreational activities than hunting (hiking,……) were allowed and because very few groups were observed within this area.
(TIF)

Figure S2 **Home ranges (fixed kernel 95% and** ***ad hoc*** **method for smoothing parameter) of 10 males fitted with GPS collars (dark blue = during hunting period; clear blue = during non-hunting period).** Plain lines correspond to the Wildlife Reserve (WR). As in the WR, hunting was prohibited within the area delimited by a dashed line. However, this area was not considered in the analysis as a protected one because all other recreational activities than hunting (hiking,……) were allowed and because very few groups were observed within this area.
(TIF)

Table S1 **Logistic regression models explaining the variation in vigilance of male mouflon based on AICc.** We generated a set of models including all combinations of the terms present in the global model and then ranked these models according to their AICc value. Only models with ΔAICc<2 were reported. Corresponding slopes were reported for covariates when included in a model. The model selected with the backward selection stepwise procedure (Table 2) was in bold font.
(PDF)

Table S2 **Logistic regression models explaining the variation in vigilance of female mouflon based on AICc.** We generated a set of models including all combinations of the terms present in the global model and then ranked these models according to their AICc value. Only models with ΔAICc<2 were reported. Corresponding slopes were reported for covariates when included in a model. The model selected with the backward selection stepwise procedure (Table 3) was in bold font.
(PDF)

## References

[1] Lima SL, Dill LM (1990) Behavioral decisions made under the risk of predation: a review and prospectus. Canadian Journal of Zoology 68: 619–640.

[2] Ludwig D, Rowe L (1990) Life-history strategies for energy gain and predator avoidance under time constraints. American Naturalist 35: 686–707.

[3] Lima SL (1998) Nonlethal effects in the ecology of predator-prey interactions. Bioscience 48: 25–34.

[4] Hipfner JM, Blight LK, Lowe R, Wilhelm SI, Robertson GJ, et al (2012) Unintended consequences: how the recovery of sea eagle *Haliaeetus* spp. populations in the northern hemisphere is affecting seabirds. Marine Ornithology 40: 39–52.

[5] Schmitz OJ, Beckerman AP, O'Brien KM (1997) Behaviorally mediated trophic cascades: effects of predation risk on food web interactions. Ecology 78: 1388–1399.

[6] Creel S, Christianson D (2008) Relationships between direct predation and risk effects. Trends in Ecology & Evolution 23: 194–201. 18308423 10.1016/j.tree.2007.12.004

[7] Boonstra R, Hik D, Singleton GR, Tinnikov A (1998) The impact of predator-induced stress on the snowshoe hare cycle. Ecological Monographs 68: 371–394.

[8] Brown JS, Laundré JW, Gurung M (1999) The ecology of fear: optimal foraging, game theory, and trophic interactions. Journal of Mammalogy 80: 385–399.

[9] Watson M, Aebischer N, Cresswell W (2007) Vigilance and fitness in grey partridges *Perdix perdix*: the effects of group size and foraging-vigilance trade-offs on predation mortality. Journal of Animal Ecology 76: 211–221. 17302828 10.1111/j.1365-2656.2006.01194.x

[10] Dimond S, Lazarus J (1974) The problem of vigilance in animal life. Brain, Behavior and Evolution 9: 60–79. 10.1159/000123655 4847593

[11] van Noordwijk AJ, de Jong G (1986) Acquisition and allocation of resources: their influence on variation in life history tactics. American Naturalist 128: 137–142.

[12] Li C, Jiang Z, Li L, Li Z, Fang H, et al (2012) Effects of reproductive status, social rank, sex and group size on vigilance patterns in Przewalski's gazelle. PLoS ONE 7: e32607. 22389714 10.1371/journal.pone.0032607 PMC3289666

[13] Frid A (1997) Vigilance by female Dall's sheep: interactions between predation risk factors. Animal Behaviour 53: 799–808.

[14] Douglas M (1971) Behaviour responses of red deer and chamois to cessation of hunting. New Zealand Journal of Science 14: 507–518.

[15] Cederna A, Lovari S (1985) The Biology and Management of Mountain Ungulates, Croom Helm, UK, chapter The impact of tourism on chamois feeding activities in an area of the Abruzzo National Park, Italy. pp. 216–225.

[16] Whittingham M, Butler S, Quinn J, Cresswell W (2004) The effect of limited visibility on vigilance behaviour and speed of predator detection: implications for the conservation of granivorous passerines. Oikos 106: 377–385.

[17] Hopewell L, Rossiter R, Blower E, Leaver L, Goto K (2005) Grazing and vigilance by Soay sheep on lundy island: influence of group size, terrain and the distribution of vegetation. Behavioural Processes 70: 186–193. 15963661 10.1016/j.beproc.2005.04.009

[18] Bednekoff P, Blumstein D (2009) Peripheral obstructions influence marmot vigilance: integrating observational and experimental results. Behavioral Ecology 20: 1111–1117.

[19] Hamilton WD (1971) Geometry for the selfish herd. Journal of Theoretical Biology 31: 295–311. 5104951 10.1016/0022-5193(71)90189-5

[20] Foster W, Treherne J (1981) Evidence for the dilution effect in the selfish herd from fish predation on a marine insect. Nature 293: 466–467.

[21] Dehn MM (1990) Vigilance for predators: detection and dilution effects. Behavioral Ecology and Sociobiology 26: 337–342.

[22] Pulliam R (1973) On the advantages of flocking. Journal of Theoretical Biology 38: 419–422. 4734745 10.1016/0022-5193(73)90184-7

[23] Lazarus J (1979) Flock size and behaviour in captive red-billed weaverbirds (*Quelea quelea*): implications for social facilitation and the functions of flocking. Behaviour 71: 127–145.

[24] Bergerud A (1974) The role of the environment in the aggregation, movement and disturbance behaviour of caribou. the behaviour of ungulates and its relation to management. IUCN Publications New Series 24: 552–584.

[25] Hunter L, Skinner J (1998) Vigilance behaviour in african ungulates: the role of predation pressure. Behaviour 135: 195–211.

[26] Laundré J, Hernández L, Altendorf K (2001) Wolves, elk, and bison: reestablishing the “landscape of fear” in Yellowstone National Park, USA. Canadian Journal of Zoology 79: 1401–1409.

[27] Childress MJ, Lung MA (2003) Predation risk, gender and the group size effect: does elk vigilance depend upon the behaviour of conspecifics? Animal Behaviour 66: 389–398.

[28] Hamel S, Côté SD (2007) Habitat use patterns in relation to escape terrain: are alpine ungulate females trading off better foraging sites for safety? Canadian Journal of Zoology 85: 933–943.

[29] Loehr J, Kovanen M, Carey J, Hogmander H, Jurasz C, et al (2005) Gender-and age-class-specific reactions to human disturbance in a sexually dimorphic ungulate. Canadian Journal of Zoology 83: 1602–1607.

[30] Garel M, Loison A, Jullien JM, Dubray D, Maillard D, et al (2009) Sex-specific growth in alpine chamois. Journal of Mammalogy 90: 954–960.

[31] Arenz C, Leger D (2000) Antipredator vigilance of juvenile and adult thirteen-lined ground squirrels and the role of nutritional need. Animal Behaviour 59: 535–541. 10715175 10.1006/anbe.1999.1345

[32] Rieucau G, Martin J (2008) Many eyes or many ewes: vigilance tactics in female bighorn sheep *Ovis canadensis* vary according to reproductive status. Oikos 117: 501–506.

[33] Rieucau G, Blanchard P, Martin JGA, Favreau FR, Goldizen AW, et al (2012) Investigating differences in vigilance tactic use within and between the sexes in eastern grey kangaroos. PLoS ONE 7: e44801. 22984563 10.1371/journal.pone.0044801 PMC3440314

[34] Sullivan KA (1988) Ontogeny of time budgets in yellow-eyed juncos: adaptation to ecological constaints. Ecology 69: 118–124.

[35] Martinetto K, Cugnasse JM, Gilbert Y (1998) La cohabitation du mouflon méditerranéen (*Ovis gmelini musimon*×*Ovis* sp.) et des touristes dans le massif du Caroux-Espinouse (Hrault). Gibier Faune Sauvage 15: 905–919.

[36] Garel M, Cugnasse JM, Maillard D, Gaillard JM, Hewison AJM, et al (2007) Selective harvesting and habitat loss produce long-term life history changes in a mouflon population. Ecological Applications 17: 1607–1618. 17913127 10.1890/06-0898.1

[37] Grignolio S, Merli E, Bongi P, Ciuti S, Apollonio M (2011) Effects of hunting with hounds on a non-target species living on the edge of a protected area. Biological Conservation 144: 641–649.

[38] Tolon V, Martin J, Dray S, Loison A, Fischer C, et al (2012) Predator-prey spatial game as a tool to understand the effects of protected areas on harvester-wildlife interactions. Ecological Applications 22: 648–657. 22611861 10.1890/11-0422.1

[39] Berger J (1978) Maternal defensive behavior in Bighorn sheep. Journal of Mammalogy 59: 620–621.

[40] Lung MA, Childress MJ (2007) The influence of conspecifics and predation risk on the vigilance of elk (*Cervus elaphus*) in Yellowstone National Park. Behavioral Ecology 18: 12–20.

[41] Fortin D, Boyce MS, Merrill EH, Fryxell JM (2004) Foraging costs of vigilance in large mammalian herbivores. Oikos 107: 172–180.

[42] Blanchard P, Fritz H (2007) Induced or routine vigilance while foraging. Oikos 116: 1603–1608.

[43] Benhaiem S, Delon M, Lourtet B, Cargnelutti B, Aulagnier S, et al (2008) Hunting increases vigilance levels in roe deer and modifies feeding site selection. Animal Behaviour 76: 611–618.

[44] Garel M, Cugnasse JM, Loison A, Gaillard JM, Vuiton C, et al (2005) Monitoring the abundance of mouflon in South France. European Journal of Wildlife Research 51: 69–76.

[45] Maublanc ML, Dubois M, Teillaud P, Cugnasse JM (1992) Effects of recreational and hunting activities on the mouflon (*Ovis ammon musimon*) population of Caroux-Espinouse. In: Spitz F, Janeau G, Gonzalez G, Aulagnier S, editors, “Ongulés/Ungulates 91”, Proceedings of the International Symposium. Toulouse, France: SFEPM-IRGM, pp. 611–615.

[46] Cabannel A, Cugnasse JM (1999) Interaction entre Renard, *Vulpes vulpes*, et Mouflon méditerranéen, *Ovis gmelini*, dans le massif de l'Espinouse (Hérault). Arvicola 1: 11–12.

[47] Cugnasse JM, Golliot E (2000) L'aigle royal *Aquila chrysatos* attaque des mouflons dans l'Espinouse. Ornithos 7: 188–190.

[48] Bon R, Dardaillon M, Estevez I (1993) Mating and lambing periods as related to age of female mouflon. Journal of Mammalogy 74: 752–757.

[49] Garel M, Cugnasse JM, Gaillard JM, Loison A, Gibert P, et al (2005) Reproductive output of female mouflon (*Ovis gmelini musimon*×*Ovis* sp.): a comparative analysis. Journal of Zoology, London 266: 65–71.

[50] Bon R, Gonzalez G, Bosch M, Cugnasse JM (1992) Ram rut-involvment in a hunted population of mouflons. Acta Theriologica 37: 63–71.

[51] Hofmann RR (1989) Evolutionary steps of ecophysiological adaptation and diversification of ruminants: a comparative view of their digestive system. Oecologia 78: 443–457. 28312172 10.1007/BF00378733

[52] Cransac N, Valet G, Cugnasse JM, Rech J (1997) Seasonal diet of mouflon (*Ovis gmelini*) : comparison of populations sub-units and sex-age classes. Revue d'Ecologie-La Terre et la Vie 52: 21–36.

[53] Marchand P, Redjadj C, Garel M, Cugnasse JM, Maillard D, et al (2013) Are mouflon *Ovis gmelini musimon* really grazers? a review of variation in diet composition. Mammal Review 43: 275–291.

[54] Cazau M, Garel M, Maillard D (2011) Responses of heather moorland and mediterranean mouflon foraging to prescribed-burning and cutting. Journal of Wildlife Management 75: 967–972.

[55] Pays O, Blanchard P, Valeix M, Chamaillé-Jammes S, Duncan P, et al (2012) Detecting predators and locating competitors while foraging: an experimental study of a medium-sized herbivore in an African savanna. Oecologia 169: 419–430. 22200851 10.1007/s00442-011-2218-3

[56] Garel M, Cugnasse JM, Hewison AJM, Maillard D (2006) Errors in age determination of mouflon in the field. Wildlife Society Bulletin 34: 300–306.

[57] Pfeffer P (1967) Le mouflon de Corse (*Ovis ammon musimon* Schreber, 1782); Position systématique, écologie et éthologie comparées. Mammalia 31 Suppl.: 1–262.

[58] Altmann J (1974) Observational study of behavior: sampling methods. Behaviour 49: 227–267. 4597405 10.1163/156853974x00534

[59] GRASS Development Team (2012) Geographic Resources Analysis Support System (GRASS GIS) Software. Open Source Geospatial Foundation URL http://grass.osgeo.org.

[60] Venables WN, Ripley BD (2002) Modern Applied Statistics with S. Fourth edition. New York, USA: Springer.

[61] Stephens PA, Buskirk SW, Hayward GD, Del Rio CM (2005) Information theory and hypothesis testing: a call for pluralism. Journal of Applied Ecology 42: 4–12.

[62] Burnham KP, Anderson DR (2002) Model selection and multimodel inference: a practical information-theoretic approach. Second edition. New York, USA: Springer.

[63] Agresti A (2002) Categorical data analysis, 2*^nd^* edition. New York: John Wiley & Sons.

[64] Le Cessie S, van Houwelingen JC (1991) A goodness-of-fit test for binary regression models, based on smoothing methods. Biometrics 47: 1267–1282.

[65] Harrell FE (2001) Regression modeling strategies: with applications to linear models, logistic regression, and survival analysis. New York, USA: Springer.

[66] Liao JG, McGee D (2003) Adjusted coefficients of dermination for logistic regression. American Statistical Association 57: 161–165.

[67] R Development Core Team (2011) R: A Language and Environment for Statistical Computing. R Foundation for Statistical Computing, Vienna, Austria. URL http://www.R-project.org/. ISBN 3-900051-07-0.

[68] Møller AP, Jennions MD (2002) How much variance can be explained by ecologists and evolutionary biologists? Oecologia 132: 492–500. 28547634 10.1007/s00442-002-0952-2

[69] Festa-Bianchet M (2003) Exploitative wildlife management as a selective pressure for the life-history evolution of large mammals. In: Festa-Bianchet M, Apollonio M, editors, Animal Behavior and Wildlife Conservation, Washington: Island Press. pp. 191–207.

[70] Milner JM, Nilsen EB, Andreassen HP (2007) Demographic side effects of selective hunting in ungulates and carnivores. Conservation Biology 21: 36–47. 17298509 10.1111/j.1523-1739.2006.00591.x

[71] Schwartz MW (1999) Choosing the appropriate scale of reserves for conservation. Annual Review of Ecology Evolution and Systematics 30: 83–108.

[72] Tolon V, Dray S, Loison A, Zeileis A, Fischer C, et al (2009) Responding to spatial and temporal variations in predation risk: space use of a game species in a changing landsape of fear. Canadian Journal of Zoology 87: 1129–1137.

[73] Dubois M, Quenette P, Bideau E, Magnac M (1993) Seasonal range use by European mouflon rams in medium altitude mountains. Acta Theriologica 38: 185–198.

[74] Dubois M, Khazraie K, Guilhem C, Maublanc ML, Le Pendu Y (1996) Philopatry in mouflon rams during the rutting season: psycho-ethological determinism and functional consequences. Behavioural Processes 35: 93–100. 10.1016/0376-6357(95)00044-5 24896022

[75] Martins AG, Netto NT, Aulagnier S, Borges A, Dubois M, et al (2002) Population subdivision among mouflon sheep (*Ovis gmelini*) ewes and ranging behaviour of rams during the rut. Journal of Zoology 258: 27–37.

[76] Smith TS, Hardin PJ, Flinders JT (1999) Response of bighorn sheep to clear-cut logging and prescribed burning. Wildlife Society Bulletin 27: 840–845.

[77] Demment MW, Van Soest PJ (1985) A nutritional explanation for body-size patterns of ruminant and non ruminant herbivores. American Naturalist 125: 641–672.

[78] Périquet S, Todd-Jones L, Valeix M, Stapelkamp B, Elliot N, et al (2012) Influence of immediate predation risk by lions on the vigilance of prey of different body size. Behavioral Ecology 23: 970–976.

[79] Togo C (1999) Vigilance behavior in lactating female alpine ibex. Canadian Journal of Zoology 77: 1060–1063.

[80] Beauchamp G (2003) Group-size effects on vigilance: a search for mechanisms. Behavioural Processes 63: 111–121. 12829305 10.1016/s0376-6357(03)00002-0

[81] Carter A, Pays O, Goldizen A (2009) Individual variation in the relationship between vigilance and group size in eastern grey kangaroos. Behavioral Ecology and Sociobiology 64: 237–245.

[82] Shackleton DM, Lovari S (1997) Classification adopted for the Caprinae survey. In: Shackleton DM, editor, Wild Sheep and goats and their relatives. Status survey and Conservation action Plan for Caprina, Gland, Switzerland and Cambridge: IUCN. pp. 9–14.

[83] Hofer D (2002) The lion's share of the hunt: Trophy hunting and conservation: A review of the legal eurasian tourist hunting market and trophy trade under cites. Technical report, Technical report, TRAFFIC Europe, Brussels, Belgium.

