# Supplementary figures and images for: Human Disturbances, Habitat Characteristics and Social Environment Generate Sex-Specific Responses in Vigilance of Mediterranean Mouflon
Source: PLoS One. 2013 Dec 30;8(12):e82960. doi: 10.1371/journal.pone.0082960 (PMC3875426; doi:10.1371/journal.pone.0082960)

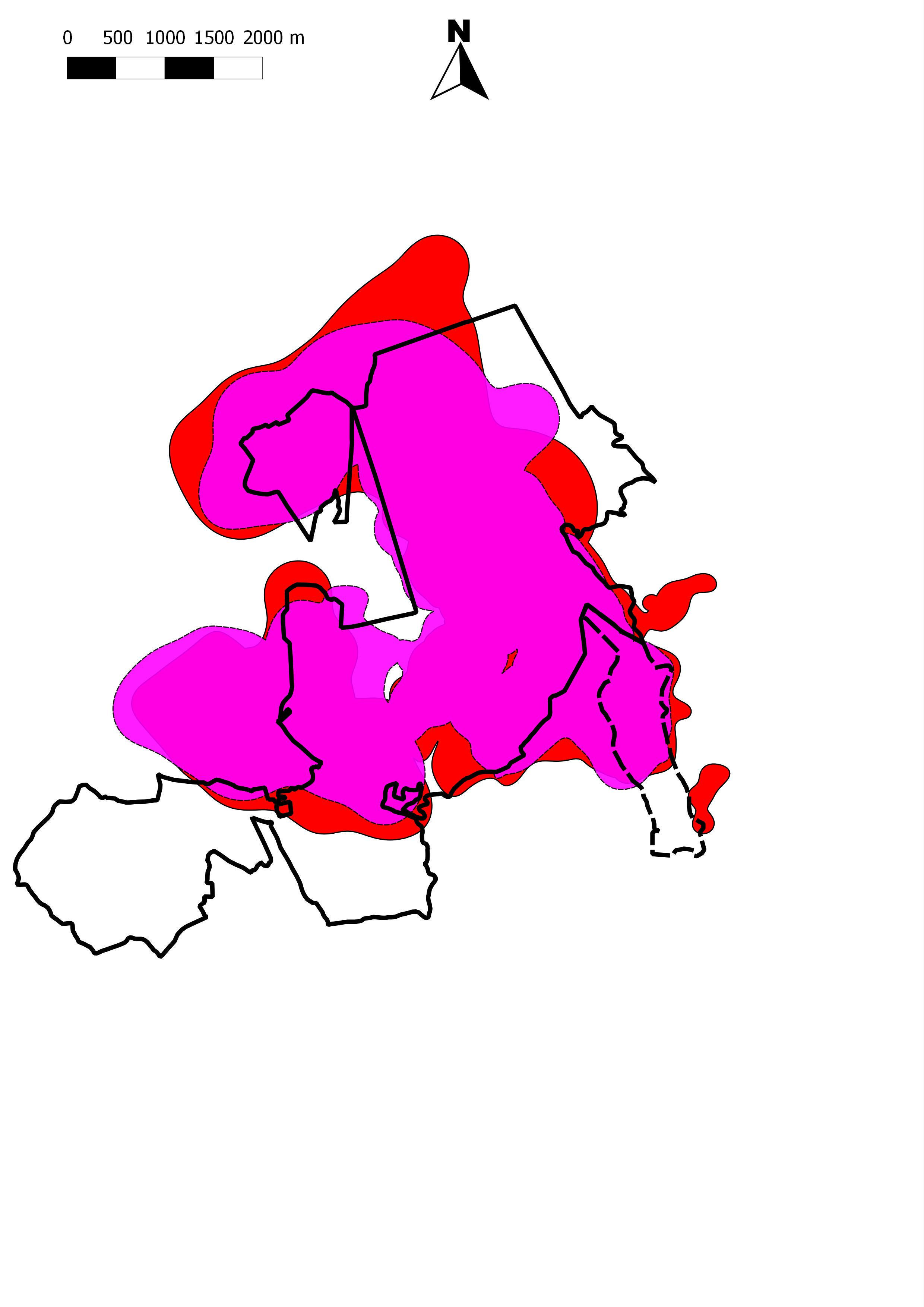

Supplement: Figure S1 — Home ranges (fixed kernel 95% and ad hoc method for smoothing parameter) of 18 females fitted with GPS collars (pink = during hunting period; red = during non-hunting period). Plain lines correspond to the Wildlife Reserve (WR). As in the WR, hunting was prohibited within the area delimited by a dashed line. However, this area was not considered in the analysis as a protected one because all other recreational activities than hunting (hiking,……) were allowed and because very few groups were observed within this area. (TIF) [file pone.0082960.s001.tif]

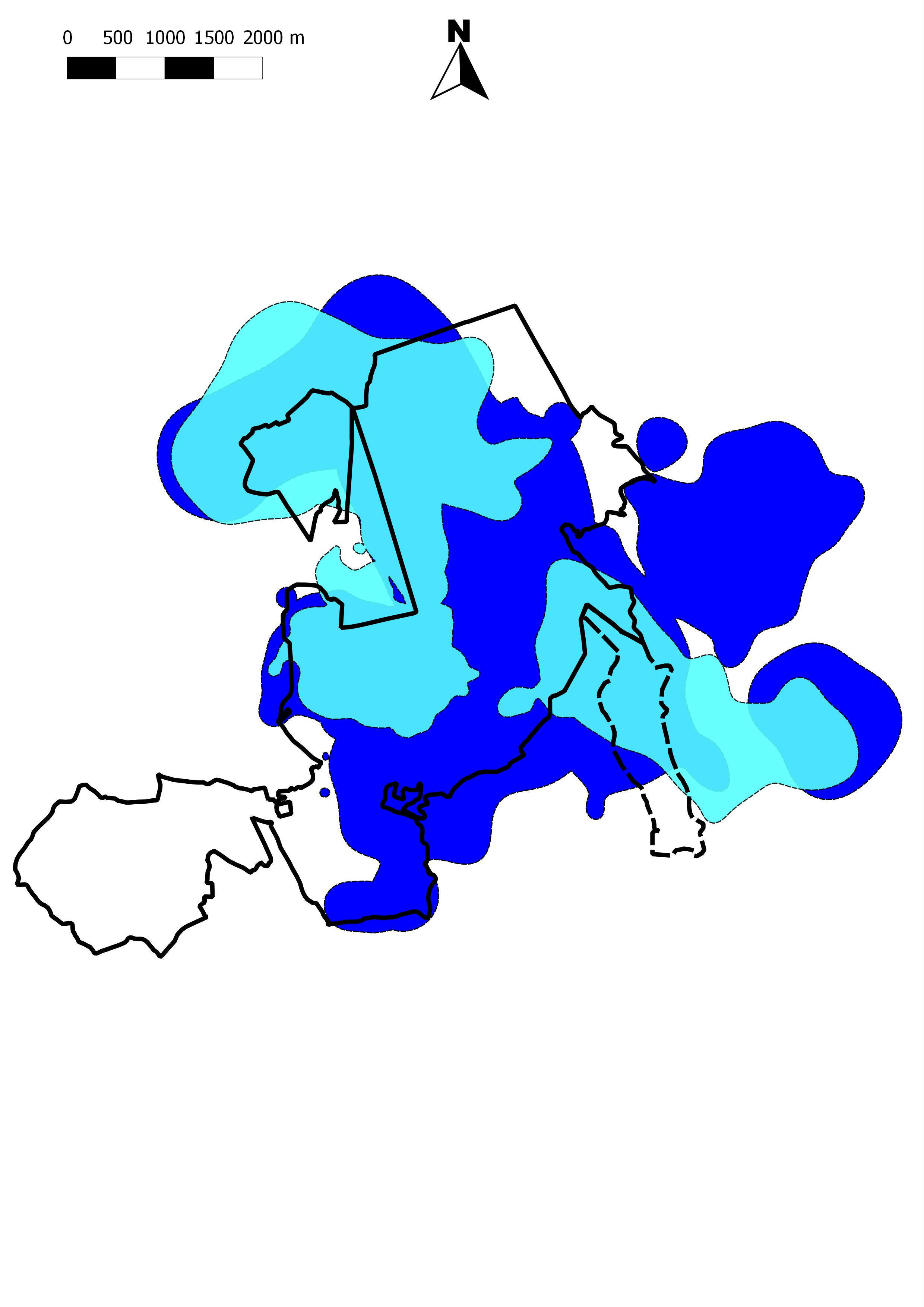

Supplement: Figure S2 — Home ranges (fixed kernel 95% and ad hoc method for smoothing parameter) of 10 males fitted with GPS collars (dark blue = during hunting period; clear blue = during non-hunting period). Plain lines correspond to the Wildlife Reserve (WR). As in the WR, hunting was prohibited within the area delimited by a dashed line. However, this area was not considered in the analysis as a protected one because all other recreational activities than hunting (hiking,……) were allowed and because very few groups were observed within this area. (TIF) [file pone.0082960.s002.tif]
