# Supplementary material for: Human Disturbances, Habitat Characteristics and Social Environment Generate Sex-Specific Responses in Vigilance of Mediterranean Mouflon
Source: PLoS One. 2013 Dec 30;8(12):e82960. doi: 10.1371/journal.pone.0082960 (PMC3875426; doi:10.1371/journal.pone.0082960)
Supplement: Table S1 — Logistic regression models explaining the variation in vigilance of male mouflon based on AICc. We generated a set of models including all combinations of the terms present in the global model and then ranked these models according to their AICc value. Only models with ΔAICc<2 were reported. Corresponding slopes were reported for covariates when included in a model. The model selected with the backward selection stepwise procedure (Table 2) was in bold font. (PDF) [file pone.0082960.s003.pdf]

Table S1. Logistic regression models explaining the variation in vigilance of male mouflon based on AICc. We generated a set of models including all combinations of the terms present in the global model and then ranked these models according to their AICc value. Only models with ΔAICc $<$ 2 were reported. Corresponding slopes were reported for covariates when included in a model. The model selected with the backward selection stepwise procedure (Table 2) was in bold font.

Models	(Intercept)	Age	Visibility	Repro compM	Hunting	Feeding	Area	Rut	Group size	Visibility×Group size	Hunting×Area	df	ΔAICc
**m1**	**-3.43**	×			×	×	×				×	**6**	**0.00**
m2	-3.28	×			×	×	×	×			×	7	0.24
m3	-3.52	×	0.003		×	×	×				×	7	1.90
