# Supplementary material for: Human Disturbances, Habitat Characteristics and Social Environment Generate Sex-Specific Responses in Vigilance of Mediterranean Mouflon
Source: PLoS One. 2013 Dec 30;8(12):e82960. doi: 10.1371/journal.pone.0082960 (PMC3875426; doi:10.1371/journal.pone.0082960)
Supplement: Table S2 — Logistic regression models explaining the variation in vigilance of female mouflon based on AICc. We generated a set of models including all combinations of the terms present in the global model and then ranked these models according to their AICc value. Only models with ΔAICc<2 were reported. Corresponding slopes were reported for covariates when included in a model. The model selected with the backward selection stepwise procedure (Table 3) was in bold font. (PDF) [file pone.0082960.s004.pdf]

**Table S2. Logistic regression models explaining the variation in vigilance of female mouflon based on AICc.** We generated a set of models including all combinations of the terms present in the global model and then ranked these models according to their AICc value. Only models with ΔAICc < 2 were reported. Corresponding slopes were reported for covariates when included in a model. The model selected with the backward selection stepwise procedure (Table 3) was in bold font.

| Models | (Intercept) | Age | Visibility | Hunting | Horn | Lambing | Feeding | Repro compF | Area | Group size | Visibility×Group size | Hunting×Area | df | ΔAICc |
|---|---|---|---|---|---|---|---|---|---|---|---|---|---|---|
| m1 | **-3.56** | × | **-0.02** | | | | | × | × | **-0.09** | | | **7** | **0.00** |
| m2 | -3.16 | × | -0.02 | × | | | | × | × | -0.09 | | | 8 | 0.54 |
| m3 | -19.62 | × | -0.02 | × | | | | × | × | -0.09 | | × | 9 | 0.58 |
| m4 | -18.70 | × | -0.01 | × | | × | | × | × | -0.10 | | × | 10 | 0.59 |
| m5 | -2.24 | × | -0.01 | × | | × | | × | × | -0.09 | | | 9 | 0.65 |
| m6 | -2.82 | × | -0.02 | | | | × | × | × | -0.10 | | | 8 | 1.20 |
| m7 | -17.69 | × | -0.02 | × | | × | × | × | × | -0.10 | | × | 11 | 1.42 |
| m8 | -1.21 | × | -0.02 | × | | × | × | × | × | -0.10 | | | 10 | 1.44 |
| m9 | -4.12 | × | | | | | | × | × | -0.08 | | | 6 | 1.50 |
| m10 | -3.51 | × | -0.02 | | × | | | × | × | -0.09 | | | 8 | 1.74 |
| m11 | -2.46 | × | -0.02 | × | | | × | × | × | -0.10 | | | 9 | 1.81 |
| m12 | -19.21 | × | | × | | × | | × | × | -0.08 | | × | 9 | 1.86 |
| m13 | -3.78 | × | -0.01 | | | | | × | × | -0.04 | -0.001 | | 8 | 1.86 |
| m14 | -18.91 | × | -0.02 | × | | | × | × | × | -0.10 | | × | 10 | 1.80 |
| m15 | -2.74 | × | | × | | × | | × | × | -0.08 | | | 8 | 1.89 |
| m16 | -3.48 | × | -0.01 | | | × | | × | × | -0.09 | | | 8 | 1.98 |
